# High-resolution native electrophoresis in-gel activity assay reveals biological insights of medium-chain fatty acyl-CoA dehydrogenase deficiency

**DOI:** 10.1038/s41598-025-24684-3

**Published:** 2025-10-23

**Authors:** Sergio Guerrero-Castillo, Alice Grün, Nicole Lewandowski, Polina Gundorova, Lisa Ela Blettenberger, Nora Constanze Laubach, Katrin Küchler, Madalena Barroso, Charlotte Uetrecht, Søren W. Gersting

**Affiliations:** 1 University Children’s Research@Kinder-UKE, University Medical Center Hamburg-Eppendorf (UKE), 20246 Hamburg, Germany; 2 German Center for Child and Adolescent Health (DZKJ), Partner Site Hamburg, University Medical Center Hamburg-Eppendorf (UKE), 20246 Hamburg, Germany; 3 CSSB Centre for Structural Systems Biology, Deutsches Elektronen Synchrotron DESY and Leibniz Institute of Virology (LIV) and University of Lübeck, 22607 Hamburg, Germany; 4 Institute of Chemistry and Metabolomics, University of Lübeck, Lübeck, Germany

**Keywords:** Fatty acid oxidation, Gel electrophoresis, Oligomer, Medium-chain acyl-CoA dehydrogenase, In-gel activity, Native electrophoresis, Metabolic disorders, Biochemistry, Enzymes, Proteins

## Abstract

Medium-chain specific acyl-CoA dehydrogenase (MCAD) is a mitochondrial homotetrameric flavoprotein that catalyzes the first step in fatty acid beta-oxidation. MCAD deficiency arises from variants that either impair enzymatic activity or destabilize interactions between subunits, leading to protein aggregation. Standard enzymatic assays measure the overall MCAD activity but cannot differentiate between tetramers and other protein forms—critical for understanding the impact of pathogenic variants on structure destabilization. In this study, we adapted a native gel colorimetric assay to quantify the activity of MCAD tetramers separately from other protein forms, providing novel insights into how pathogenic variants affect MCAD structure and function. The assay showed a linear correlation between protein amount and enzymatic activity for octanoyl-CoA, a physiological MCAD substrate. Applying this method to clinically relevant MCAD variants allowed us to distinguish subtle differences in protein shape, enzymatic activity, and FAD content, offering profound implications for understanding the molecular basis of MCADD. This methodology can be extended to analyze variants in other acyl-CoA dehydrogenase family members—such as glutaryl-CoA, isovaleryl-CoA or short-chain fatty acyl-CoA dehydrogenases—that are implicated in disorders of fatty acid and amino acid metabolism.

**Supplementary Information:**

The online version contains supplementary material available at 10.1038/s41598-025-24684-3.

## Introduction

The first step in mitochondrial fatty acid beta-oxidation is the dehydrogenation from C-2 and C-3 of a straight-chain fatty acyl-CoA thioester, resulting in the formation of 2-enoyl-CoA with a *trans* double bond, and the reduction of the cofactor, flavin adenine dinucleotide (FAD)^1^. This reaction is catalysed by several proteins with different specificities towards the acyl chain length. Medium-chain specific acyl-CoA dehydrogenase (MCAD; EC: 1.3.8.7), encoded by *ACADM*, has higher specificity for substrates with acyl chains of six and eight carbon atoms, although it can oxidize acyl-CoA thioesters from C4 to C16^2^. *ACADM* encodes a 46.6-kDa precursor protein of 421 amino acid residues, including an N-terminal mitochondrial-targeting sequence^3^. This precursor is cleaved at position 25 by the mitochondrial targeting peptidase, rendering a mature protein of 43.6 kDa with alpha helix domains at the N- and C-termini and a middle β-domain^4^. After its import and maturation in the mitochondrial matrix, it forms a homotetramer^5–7^ with a theoretical molecular mass of 177.7 kDa, containing one FAD per monomer.

Homozygous or compound heterozygous variants in *ACADM* lead to MCAD deficiency (MCADD; OMIM #201450)^8^. MCADD is characterized by medium-chain dicarboxylic acidemia, low carnitine levels and intolerance to prolonged fasting, as ketone body formation is fuelled by and requires optimal functioning of fatty acid beta oxidation. Therefore, individuals with MCADD must avoid prolonged fasting to decrease the risk of hypoketotic hypoglycemic coma due to impaired energy conversion via oxidation of fatty acids and ketone bodies^9^. MCADD is diagnosed early in newborn screenings by profiling acylcarnitines using tandem mass spectrometry (MS/MS), in which characteristic elevations in hexanoyl-, octanoyl-, and decanoyl-carnitine are detected^10^.

The enzyme activity of MCAD variants has generally been determined by spectrofluorometric assays, monitoring the reduction of the physiological catalytic interactor, the electron transfer flavoprotein complex^11,12^, or spectrophotometric techniques, using artificial electron acceptors such as ferricenium hexafluorophosphate^13^ or 2,6-dichlorophenolindophenol (DCPIP) in combination with phenazine methano- or ethano-sulfate^14^. Other methods include high-pressure liquid chromatography (HPLC) coupled with UV detection or mass spectrometry (MS) to quantify the oxidized product octenoyl-CoA, or alternative products such as cinnamoyl-CoA, the product of the dehydrogenation of the alternative substrate phenylpropionyl-CoA^15^. In addition, MCAD activity can be monitored in vivo by a breath test method using stable isotopes, ^13^C-octanoic acid or ^13^C-phenylpropionic acid^16^. These techniques offer unique advantages such as robustness, reproducibility, high sensitivity, and the ability to measure MCAD activity in vivo. However, these activity measurements do not allow distinguishing the activity of tetramers from other protein forms, such as aggregates likely generated by pathogenic variants. Particularly, it has been shown that variants altering MCAD folding may potentially destabilize its quaternary structure, resulting in either protein aggregation or fragmentation of the tetramer into lower molecular mass forms^17^. However, the extent to which these irregular conformations of MCAD contribute to the overall enzymatic activity remains unknown.

Methods for determining the enzymatic activity in a gel offer the advantage of separating proteins by their size, allowing to differentiate the activity, stoichiometry and abundance of each protein form. In-gel activity staining procedures have been previously applied for determination of various mitochondrial respiratory chain enzyme complexes^18^. Moreover, high-resolution separation of oxidative phosphorylation complexes by blue- and clear-native electrophoresis facilitates discerning between several supercomplexes of varying composition and stoichiometry^19^. Although these assays have mostly been applied to study multi-subunit, macromolecular complexes and supercomplexes^20–22^, methods to determine in-gel enzymatic activities have also been developed for smaller proteins with less complexity, including nicotinamide adenine dinucleotide kinase^23^ and glucan phosphatases^24^. In-gel enzymatic activity assays of oxidoreductases have been established by coupling substrate oxidation with reduction of an electron acceptor with colorimetric properties such as tetrazolium salts, commonly used for instance in histology staining methods.

Here, we adapted an assay to determine in-gel the enzymatic activity of MCAD, coupling the oxidation of octanoyl-CoA with the reduction of nitro tetrazolium blue chloride after high-resolution clear native gel electrophoresis. We applied the assay to assess the activity of MCAD variants as purified recombinant proteins or in mitochondrial-enriched fractions from cell homogenates. Last, we explored the potential of the assay to be extended to other members of the acyl-CoA dehydrogenase family, such as short-chain fatty acyl-CoA dehydrogenase, isovaleryl-CoA dehydrogenase, or glutaryl-CoA dehydrogenase.

## Results

### Adapting a fast and sensitive colorimetric in-gel assay to determine the activity of MCAD

**Fig. 1:**
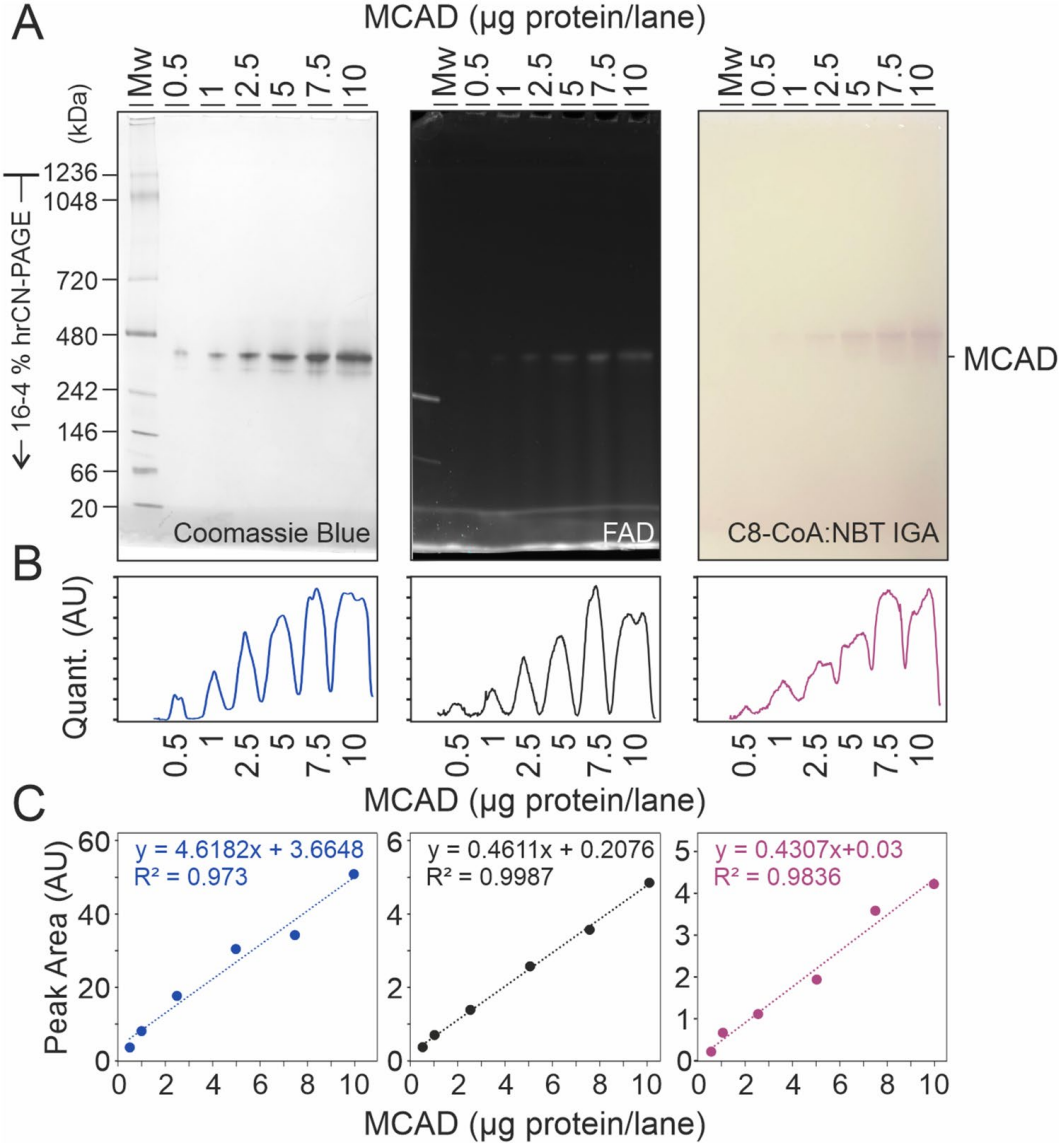
Octanoyl-CoA:NBT oxidoreductase in-gel activity of human recombinant MCAD WT. (**A**) High-resolution clear native (hrCN) gels after electrophoretic separation of human recombinant MCAD WT at pH 7.0. From left to right: Coomassie blue stained gel; Intrinsic FAD fluorescence; and C8-CoA:NBT oxidoreductase in-gel activity. (**B**) Quantification profiles of the main oligomer band. (**C**) Integrals from the quantification peaks were plotted as a function of the protein amount per lane. This figure shows a representative gel (n = 3).

Pathogenic variants causing metabolic disorders can either affect the enzymatic activity, or the protein folding, altering the overall structure and leading to protein degradation. In the case of multimeric proteins such as MCAD, disrupting interactions between subunits often results in tetramer fragmentation or in protein aggregation^17,25^. Given the limitations of standard assays in distinguishing between different forms of MCAD, we adapted an in-gel colorimetric assay tailored to determine MCAD enzymatic activity, while providing qualitative insights into the structural diversity of the enzyme. After separation of recombinant MCAD by high-resolution clear native PAGE (hrCN-PAGE), we stained the gel in a solution containing the physiological MCAD substrate, octanoyl-CoA, as a reductant, and nitro blue tetrazolium chloride (NBT) as oxidizing agent, which forms an insoluble, purple-colored diformazan precipitate^18^. The formation of purple bands at apparent mass ranges between 240 and 480 kDa became visible after 10–15 min incubation of the gel in the reaction mixture. To test whether the in-gel activity correlated with the amount of purified protein and with the FAD content, increasing amounts of human recombinant MCAD WT were loaded on a 4–16% high-resolution clear native gel (Fig. 1A). In addition to the main band, a second, less intense band was visible at an apparent lower molecular mass range, suggesting the presence of a different MCAD species, which was also active. Densitometric analysis of the predominant band (Fig. 1B) showed linear correlations for protein amount, FAD content and in-gel activity (Fig. 1C), indicating that the assay was sensitive enough to quantify the activity of even less than 1 µg of protein.

### In-gel activity of MCAD variants

**Fig. 2:**
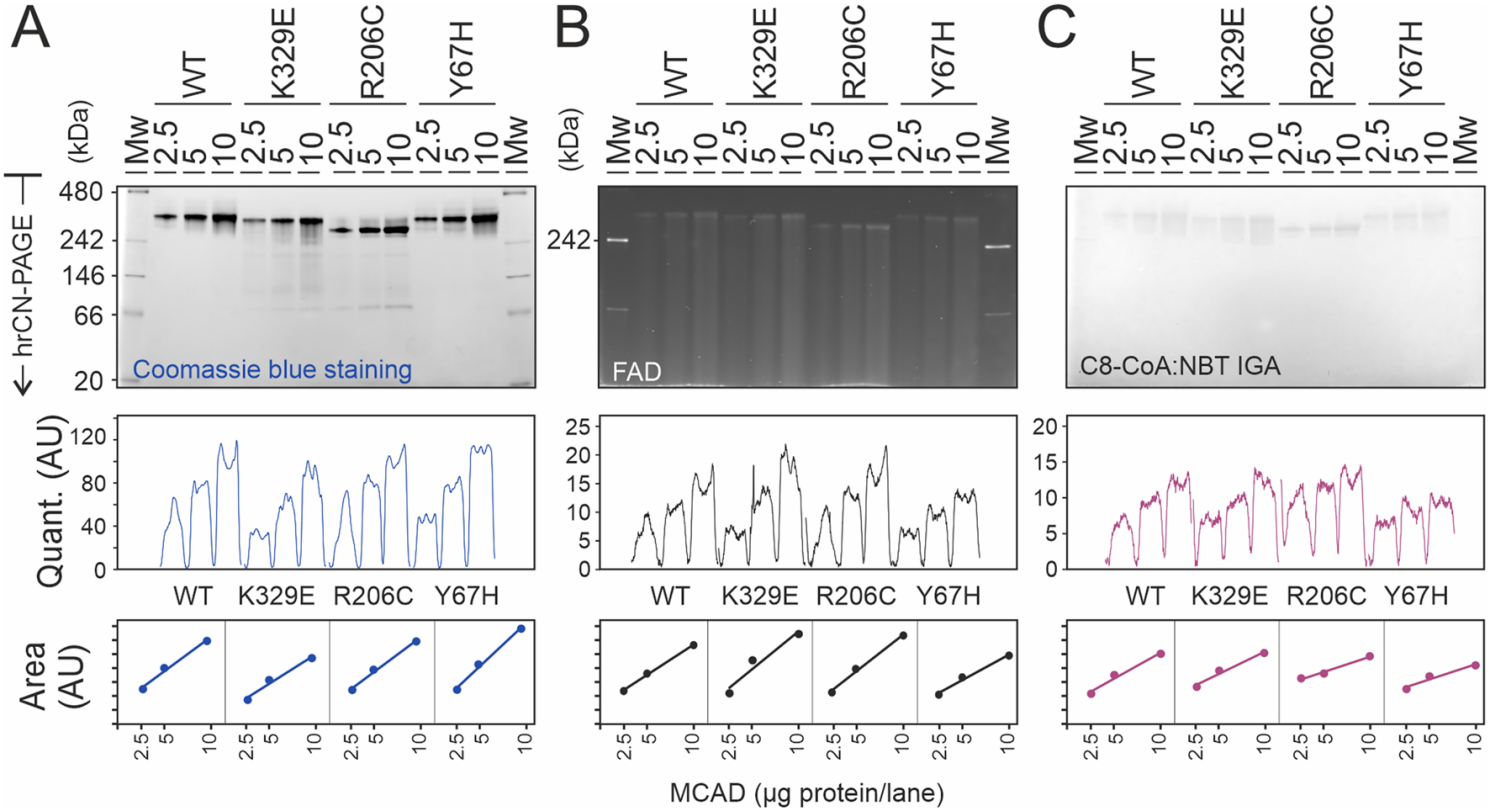
In-gel activity and FAD content of MCAD protein variants. Human recombinant MCAD wild type and three variants were separated by hrCN-PAGE at pH 7.0. (**A**) hrCN gel stained with Coomassie blue. (**B**) FAD content determined by intrinsic fluorescence. (**C**) Octanoyl-CoA:NBT oxidoreductase in-gel activity. (**A**–**C**) Gels were documented (upper panels), pixel density was quantified using ImageJ (middle panels), and the peak areas were calculated using Origin (bottom panels).

We next explored the impact of specific missense variants on the enzyme oligomeric state and function. We applied the in-gel assay to determine the activity of MCAD variants of clinical relevance using human recombinant purified protein. To this end, we selected three previously studied MCAD variants representative of each of the three protein subdomains^17,25^. These variants included p.Y67H, localized at the N-terminal alpha helix domain; p.R206C, localized in the middle beta domain; and p.K329E, a prevalent variant in a C-terminal alpha helix localized at the interface between MCAD subunits. These variants are known to destabilize the conformation of the protein rather than impairing the catalytic activity of the enzyme^17^. To determine the in-gel activity of MCAD variants and to correlate it with the amount of protein and FAD content, we loaded increasing amounts of protein in parallel gels and quantified the main bands by densitometry (Fig. 2A). The amount of protein was determined from the gel stained with Coomassie blue, in which MCAD WT and variant Y67H presented one predominant band below 480 kDa and a series of bands of very low abundance slightly above and below, while variants K329E and R206C, in addition to the main band, showed less intense bands at much smaller molecular mass ranges, suggesting fragmentation of the tetramers in these variants. A replicate gel was used for both, determination of FAD content (Fig. 2B) and for octanoyl-CoA:NBT oxidoreductase in-gel activity (Fig. 2C). The amount of FAD bound to the predominant MCAD species in the three variants was similar to the WT, suggesting that these variants do not affect FAD binding. However, this quantification is limited by the fact that a fraction of FAD seem to have detached from the protein during electrophoresis and migrated to the front. In addition, in the variants K329E and R206C, the redox state of the FAD was fully reduced as indicated by the absorbance ratio at 278/450 nm (Supplementary Fig. S1A), hampering FAD content quantification. In-gel activity staining showed activity bands with similar intensity in all variants above 240 kDa. However, the MCAD species observed at lower molecular mass ranges in variants K329E and R206C were not active, in agreement with the lower enzymatic activity determined spectrophotometrically (Supplementary Fig. S1B). Interestingly, the main band of MCAD tetramers in variant R206C in the gels presented in Fig. 2 did not appear at the same position as the WT or the two other MCAD variants. It migrated 5 mm more than the WT at an apparent lower molecular mass, although no shift was observed after separation by SDS-PAGE (Supplementary Fig. S1C), indicating that the monomeric molecular mass was the same for all variants. Consistent with the separation pattern in clear native gels, the apparent molecular mass shift of variant R206C was also observed after separation by blue-native electrophoresis (Supplementary Fig. S1C), where proteins were separated mostly according to their molecular mass, since the Coomassie blue dye present in the sample and cathode buffers confers a negative charge to the proteins. These observations suggest that variant R206C induces a conformational change that may modify the intrinsic charge, hydrophobicity or the overall arrangement of the tetramer towards a more compact conformation.

### Investigating MCAD variants in eukaryotic systems

**Fig. 3:**
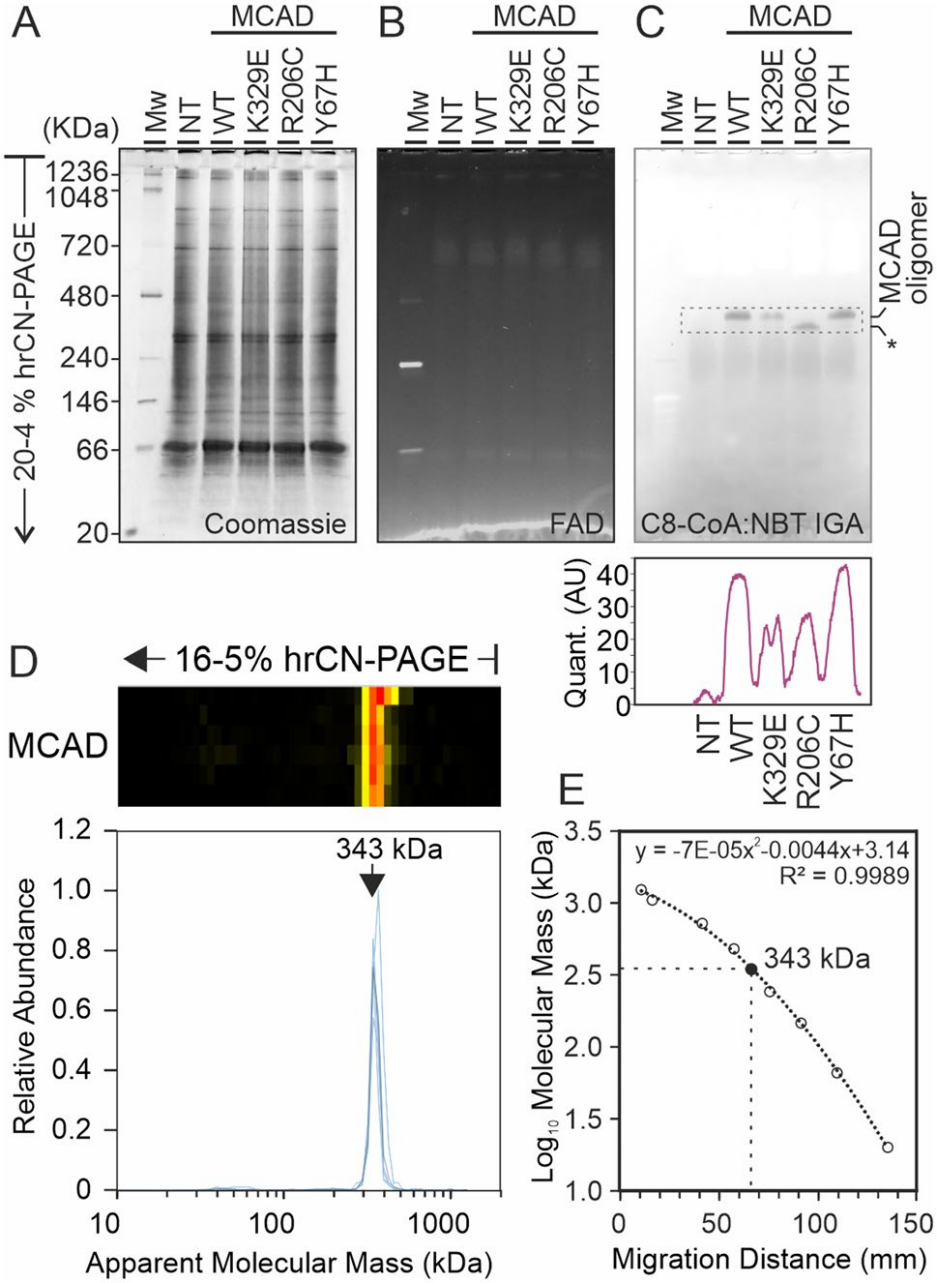
Overexpressed MCAD variants, and endogenous MCAD oligomers in eukaryotic systems. (**A**) Mitochondrial fractions of COS-7 cells overexpressing MCAD wild type and the three variants were separated by hrCN-PAGE at pH 7.0. Duplicate gels were used, one for Coomassie blue staining (left panel) and the other for FAD content (middle panel) and subsequently C8-CoA:NBT in-gel activity (right panel). Quantification of the in-gel activity bands (right bottom panel). NT: Non-transfected cells. * MCAD oligomer with lower apparent molecular mass. (**B**) Abundance heatmap and migration profiles of MCAD from six human skin fibroblast cell lines after separation by hrCN-PAGE. Individual profiles are shown in blue, average profile in gray. (**D**) Molecular mass calibration of protein NativeMark standards and interpolation of the predominant MCAD peak.

To further characterize the effects of the variants on the conformation of MCAD tetramers, we overexpressed these variants in a eukaryotic model system. This environment can mimic the physiological conditions better than the purified recombinant protein, since, in living cells, there are several factors that modulate or respond to protein misfolding, such as molecular chaperones or solvent conditions. These factors also influence the amount of active MCAD available in patient cells. To this end, we transfected COS-7 cells to overexpress MCAD variants. After separation of COS-7 cell lysates by hrCN-PAGE, transfected cells presented one additional band at ca. 340 kDa that was not observed in non-transfected cells (Fig. 3A). However, the intrinsic FAD fluorescence of MCAD was too low to be determined with the settings used, since no evident fluorescence was detected at this molecular mass range. In turn, we observed faint bands at higher and lower molecular mass ranges, most likely corresponding to other endogenous FAD-containing dehydrogenases of higher abundance (Fig. 3B). Nevertheless, in-gel C8-CoA:NBT oxidoreductase activity staining revealed the formation of active MCAD tetramers (Fig. 3C) with the same separation patterns as the recombinant proteins expressed in *E. coli*, including the apparent molecular mass shift observed in variant R206C. This result indicates that the protein conformation and stoichiometry was the same in both overexpression systems.

Next, to investigate the conformation and oligomeric state of MCAD in human samples with endogenous levels of MCAD expression and physiological FAD concentrations, we analysed a proteomics data set of mitochondrial enriched fractions from human skin fibroblasts separated by hrCN-PAGE. The entire gel lanes were fractionated, digested with trypsin and the peptide solutions were subjected to liquid chromatography- tandem mass spectrometry. In these samples, MCAD showed a single peak at 340 kDa (Fig. 3D), as calculated by interpolation using the migration distance of molecular mass standards (Fig. 3E). The apparent molecular mass is higher than the expected mass of the tetramer. However, this result confirms that the protein conformation and stoichiometry observed in transfected cells was the same as the protein expressed in human samples at endogenous levels.

### Analysis of MCAD variants by gel filtration, IEF and differential scanning fluorimetry

**Fig. 4:**
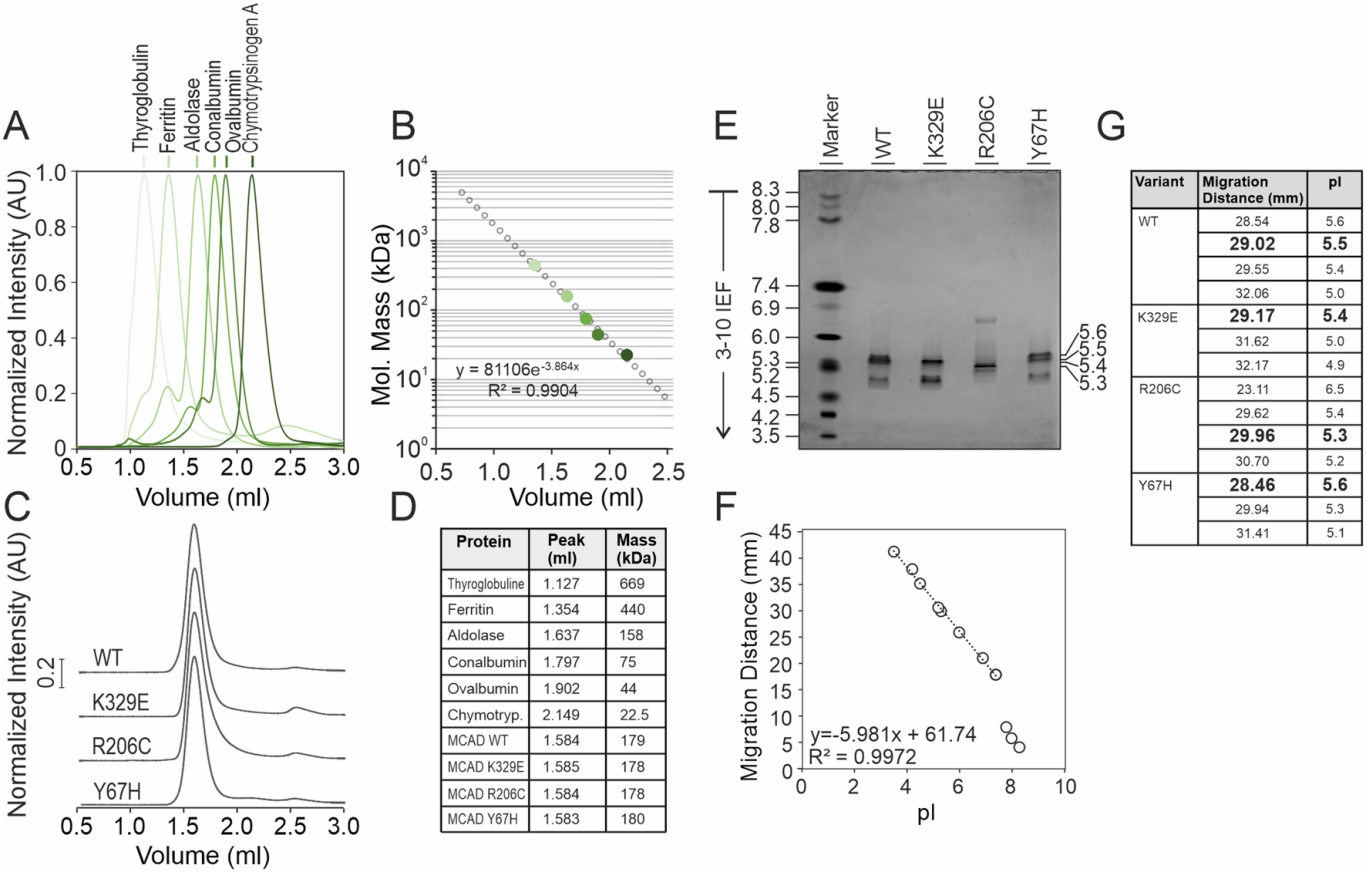
Size exclusion chromatography analysis of MCAD variants. (**A**) Separation of the protein standards used for molecular mass calibration. (**B**) Calibration curve plotting the logarithms of the molecular masses of the standards as a function of their elution volumes. (**C**) Separation of human recombinant MCAD WT and variants, determined by absorbance at 280 nm, see Experimental Procedures for method details. (**D**) Interpolation of elution volumes and estimated molecular masses of MCAD variants. (**E**) Isoelectric focusing of MCAD variants using 3–10 IEF gels followed by Coomassie blue staining. (**F**) Migration distance of isoelectric focusing markers as a function of the isoelectric point. The three most basic proteins stained were excluded from the linear regression to create the calibration curve. (**G**) Migration distance and isoelectric points of isoforms detected for each MCAD variant.

To further explore the apparent mass shift in variant R206C, and given the discrepancy on the molecular mass estimation of the WT MCAD tetramer by hrCN-PAGE, we used size exclusion chromatography as a more reliable method to characterize the mass of the tetramers. Elution volumes of commercial molecular mass standards for gel filtration were used for mass calibration (Fig. 4A-B). The separation patterns of MCAD presented a single peak with a maximum at the same position in all variants (Fig. 4C). By interpolating the elution volumes in the calibration curve, we calculated the molecular mass of MCAD WT and variants (Fig. 4D), which all fit with the theoretical mass of the tetramer (177 kDa), indicating that the apparent mass shift observed in hrCN gels for variant R206C was not due to a different stoichiometry or molecular mass.

Next, to investigate whether the intrinsic charge of MCAD tetramers contributes to the apparent mass shift observed in hrCN gels, we experimentally determined the isoelectric points (pI) of MCAD WT and three variants using isoelectric focusing gels (Fig. 4E–G). Notably, alongside a prominent band with a pI of approximately 5.5, we identified two to three additional bands for each MCAD variant, which may represent distinct conformations of the tetramer. However, as all observed isoelectric points were within the acidic range, it seems unlikely that intrinsic charge alone accounts for the atypical separation patterns observed in hrCN gels.

To test whether the ionic strength plays a role on the abnormal separation of MCAD by hrCN-PAGE, we employed different concentrations of 6-aminohexanoic acid (AHA) in the solubilization buffer. The lack of AHA affected protein solubility and the sharpness of the bands but did not influence the mobility of MCAD (Supplementary Fig. S2).

Next, we evaluated the hydrophobicity of recombinant MCAD variants, as increased hydrophobicity in vitro could enhance binding to the negatively charged detergent deoxycholate during hrCN-PAGE. This interaction may partially account for the distinct migration behavior observed for the R206C variant compared to the wild-type (WT) and the other two variants. To assess hydrophobicity, we performed differential scanning fluorimetry and compared baseline fluorescence levels across the variants. R206C exhibited a marked elevation in baseline fluorescence relative to both WT and K329E, while Y67H showed reduced baseline fluorescence compared to WT (Supplementary Fig. S3).

### Top-down native mass spectrometry analysis of recombinant MCAD.

To further explore the distinct potential conformations of MCAD tetramers, we analysed purified human recombinant MCAD by native MS. Native top-down MS is a powerful tool for characterizing intact proteins and macromolecular assemblies and provides information on the quaternary structure and stoichiometry of proteins^26^. As the protein was purified in a buffer with a high salt concentration, first, it was necessary to replace the buffer with one compatible with native MS, such as ammonium acetate, and evaluate whether the MCAD conformation was not altered by exchanging the buffer prior to the analysis. Supplementary Fig. S4A shows the native separation by BN- and hrCN-PAGE of fresh and frozen/thawed human recombinant MCAD tetramers in the buffer used for purification (SEC buffer) and in ammonium acetate buffer. It was observed that after buffer replacement and the additional freezing/thawing cycle MCAD tetramers migrated in native gels at the same position in all four conditions tested and preserved its activity, as shown by C8-CoA: NBT in-gel oxidoreduction. It was noticed, however, that the apparent molecular mass of the main MCAD band varied depending on the type of native electrophoresis used. In BN-PAGE, the tetramer was separated at an apparent lower molecular mass than in the hrCN counterpart, even though all protein standards used for the molecular mass calibration migrated to the same position in both types of native gels. According to independent molecular mass calibrations for each type of gel, MCAD migrated at an apparent molecular mass of ca. 350 kDa and 260 kDa, in hrCN-PAGE and BN-PAGE, respectively (Supplementary Fig. S4B), suggesting that the intrinsic charge of the protein could partially affect the electrophoretic mobility and that the migration distance cannot be used to estimate the molecular mass or the oligomeric state of MCAD.

**Fig. 5:**
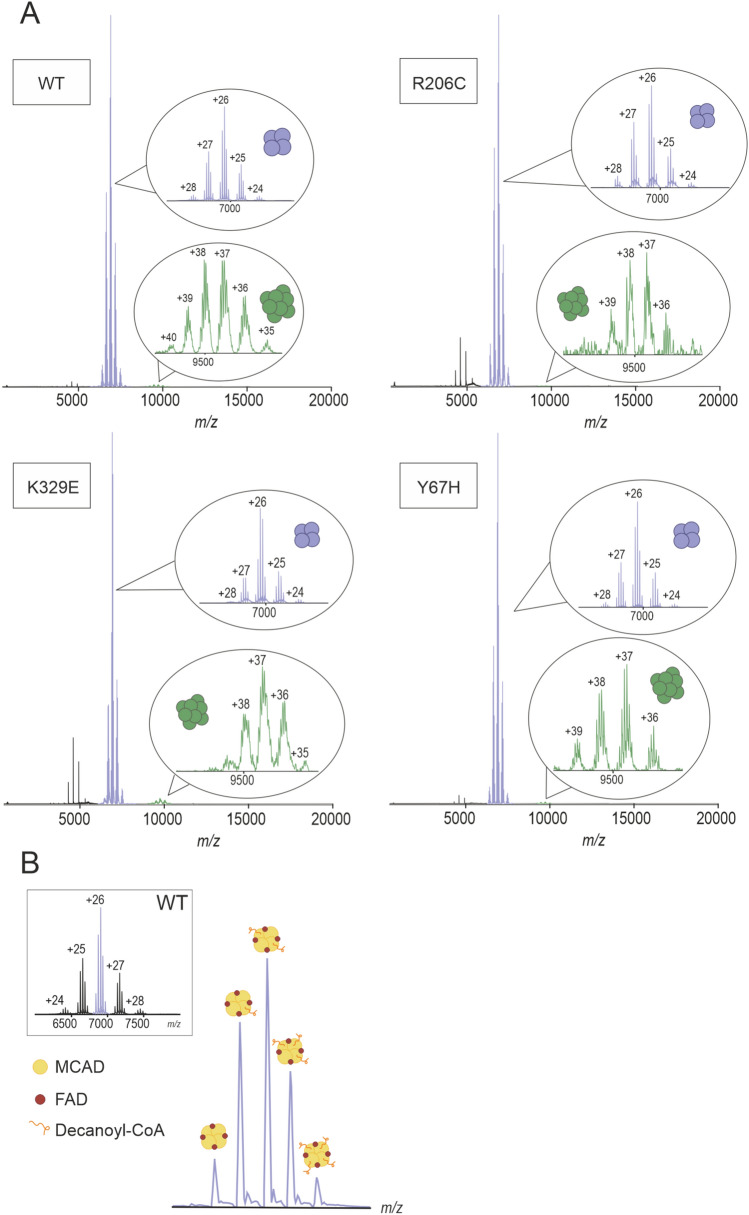
Native mass spectrometry analysis of human recombinant MCAD variants. (**A**) Representative mass spectrum of MCAD variants. Insets in each panel display charge states of the tetrameric (in blue) and of the octameric (in green) conformations. (**B**) Zoom in of the MCAD WT tetramer 26 + charge state with an increasing number of bound decanoyl-CoA molecules. Inset shows the MCAD WT tetramer.

After testing that the MCAD tetramer was stable in ammonium acetate buffer, the protein was analysed by native MS. The full spectrum showed peaks at approximately 7000 m*/z* that corresponded to charge states from + 24 to + 28 of the MCAD WT tetrameric conformation with well resolved fine structure corresponding to masses ranging from 178,010 to 181,690 Da (Fig. 5A, Supplementary Table 1). In addition, a second group of peaks at ca. 9500 m*/z* with charge states from + 35 to + 40 were observed, which corresponded to an octamer of approximately 353 kDa (Fig. 5A). However, such signals were much less intense than those of the tetramer. In order to verify that the peaks are indeed from tetramers and octamers, MS/MS was performed on a Q-TOF 2. Single peaks corresponding to the tetramers and octamers were isolated and fragmented. MS/MS analysis confirmed detaching of one subunit from the tetramers and octamers during fragmentation (Supplementary Fig. S5A-B). We further explored and compared the native mass spectra of recombinant MCAD variants K329E, R206C and Y67H. In all spectra, the predominant peaks belonged to the tetrameric species and presented an equivalent fine structure with the same charge distribution for all variants. In addition, very low intensity peaks corresponding to an octameric conformation was observed in all variants. Interestingly, the spectra of all variants indicated different MCAD tetrameric species with an increase in molecular mass of 920 Da, matching the molecular mass of an acyl-CoA with a 10-C chain length (Supplementary Tables 1 and 2). This analysis revealed, in addition to FAD binding, MCAD species containing up to four 920-Da molecules (Fig. 5B). The fine structure revealed that variant R206C binds only three FAD molecules per tetramer instead of four, as the other variants (Supplementary Table 1), providing a plausible explanation for the different species of MCAD tetramers observed by hrCN-PAGE and IEF. This mass difference, however, is not sufficient to explain the mass shift observed by hrCN-PAGE separation for this variant. Native MS indicated that the R206C variant did not cause any large change in the mass of the tetramer, except for the single residue exchange due to the point mutation. The lack of one FAD molecule in the R206C tetramer decreased the proportion of decanoyl-CoA molecules bound to this protein variant in comparison to the WT and to the other two variants (Supplementary Tables 1 and 2). This could reflect adaptive or maladaptive responses to structural instability induced in the variants, suggesting a potential mechanism for the diverse clinical presentations of MCADD.

### Expanding the in-gel activity determination to other acyl-CoA dehydrogenases

**Fig. 6:**
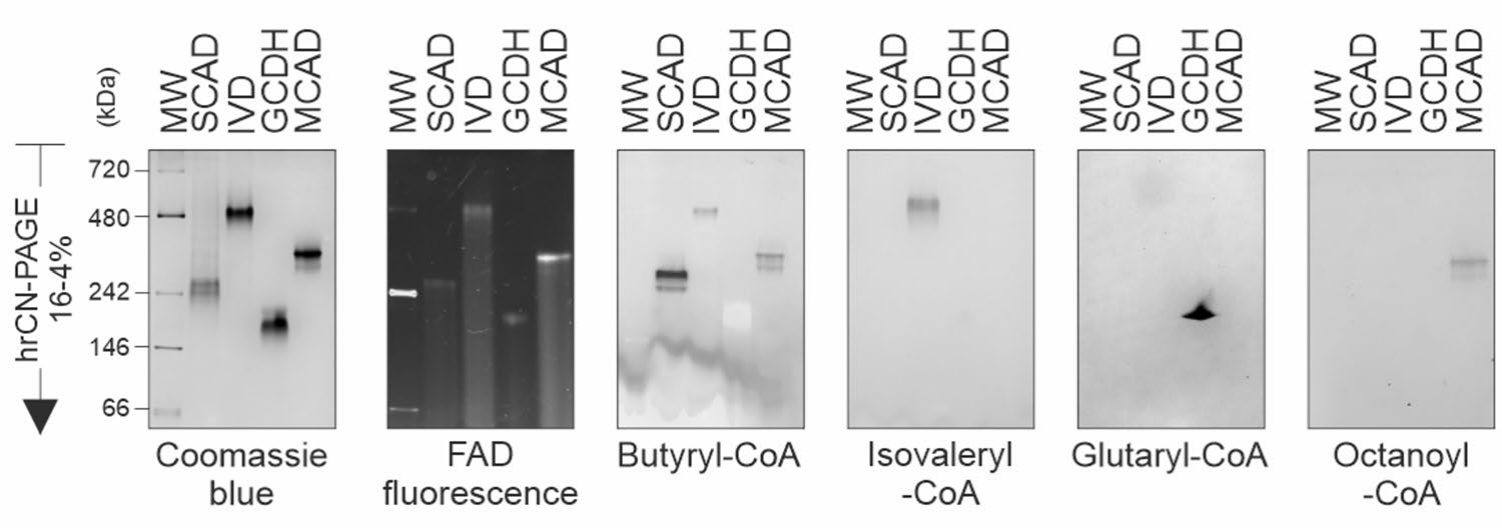
In-gel activity staining of other ACAD family members. High-resolution clear native gels showing electrophoretic separation of human recombinant SCAD, IVD, GCDH and MCAD. From left to right: Coomassie blue stained gel; Intrinsic FAD fluorescence; and acyl-CoA:NBT oxidoreductase in-gel activities with their respective substrates.

The in-gel enzymatic assay developed for MCAD can be readily extended to other members of the acyl-CoA dehydrogenase family by replacing the acyl-CoA substrate used as a reductant agent in the redox reaction, matching the substrate specificity for each enzyme. This assay can be performed after separation by blue or by clear native electrophoresis. As an example, we tested the in-gel activities of three other acyl-CoA dehydrogenases: small-chain acyl-CoA, isovaleryl-CoA and glutaryl-CoA dehydrogenases, SCAD, GCDH and IVD, respectively. MCAD was also included for comparison purposes. The recombinant proteins were separarted by hrCN-PAGE and parallel gels were incubated with the artificial electron acceptor, NBT, and with the respective substrates: butyryl-CoA, isovaleryl-CoA, glutaryl-CoA and octanoyl-CoA, resulting in specific staining (Fig. 6). Even though, the four dehydrogenases are tetramers formed by subunits of similar mass (between 41.6 and 43.6 kDa) (Supplementary Fig. S6A), except for GCDH, they did not migrate at the expected mass range. In particular, IVD showed very low mobility in the hrCN gels, probably due to its high isoelectric point in comparison to the other dehydrogenases tested (Supplementary Fig. S6B). Together, these results indicate that this assay can be further used to study disease-related variants in other dehydrogenases from this protein family.

## Discussion

The catalytic activity of MCAD is traditionally assessed using rapid equilibrium kinetic assays where enzymatic rates are measured with spectrophotometric^14^ or spectrofluorometric^27^ methods as a function of substrate concentration. However, these methods do not distinguish between the activity of MCAD tetramers and abnormal subcomplexes or aggregates generated by pathogenic *ACADM* variants*.* As a result, the contribution of different protein conformations to the total MCAD activity remained elusive. To address this limitation, we implemented an in-gel colorimetric assay to determine the enzymatic activity of MCAD after separation by high-resolution clear native gel electrophoresis. This method enables resolution of individual protein conformations within samples with heterogeneous MCAD species.

The differences in enzymatic activity between wild type and MCAD variants observed with this method were comparable to the ones determined previously using spectrophotometric techniques^25^. Notably, our approach offers the advantage of simultaneously assessing total protein amount, FAD content and enzymatic activity in a single run. However, one inherent limitation of this semi-quantitative method is its inability to provide absolute quantification of protein abundance. Consequently, band intensities should be interpreted relative to a control condition or, in the case of protein variants, compared directly to a wild-type enzyme analysed concurrently under identical conditions.

An additional consideration is the atypical migration behaviour of MCAD observed in native gel electrophoresis. In both blue-native and high-resolution clear-native PAGE, the predominant wild-type MCAD band exhibited abnormal separation patterns, corresponding to an apparent molecular mass of approximately 260 kDa and 350 kDa, respectively—substantially higher than the expected mass of the tetramer. It is important to recognize that in native gels, separation of soluble proteins does not depend solely on the molecular mass of the proteins, as the intrinsic charge, globular shape and detergent binding capacity may also influence their electrophoretic mobility^28^. In contrast, size exclusion chromatography of recombinant MCAD variants revealed a single peak corresponding to the expected tetrameric mass, based on the crystallographic structure of MCAD WT, PDB 1EGE^4^. Structural models of MCAD, including those of human and pig proteins have long established that MCAD assembles into a homotetramer with a calculated molecular mass of approximately 177.54 kDa. Each monomer within the tetramer harbors a single molecule of FAD as a covalently bound prosthetic group. From those structural models the catalytic residues and substrate binding sites, not only for the fatty acyl-CoA but also for the endogenous electron acceptor, ETF complex, were identified^29,30^.

The observed abnormal separation of the main MCAD band at a higher apparent molecular mass—suggestive of an octamer—along with additional bands differing by the monomer mass, might lead to misinterpretations of the stoichiometry of MCAD. This irregular apparent molecular mass was consistently seen across various kinds of samples, including recombinant protein expressed in prokaryotic and eukaryotic systems, as well as endogenous MCAD in mitochondria from human fibroblasts. Despite the presence of deoxycholate in the cathode buffer, the separation patterns in clear native gels was atypical. These irregularities likely result from influences such as protein intrinsic charge, redox state, globular shape or interactions with detergents that influenced their migration behaviour^28^. In this regard, the slight differences in the observed isoelectric points of MCAD variants could also partially add to the irregular migration patterns. For instance, the variant with the lowest isoelectric point, R206C, was the protein variant migrating the farther. However, the atypical migration pattern of the R206C variant compared to the other three MCAD proteins is more plausibly attributed to differences in hydrophobicity. The increased hydrophobic character of R206C may enhance its interaction with deoxycholate, thereby imparting a stronger negative charge and promoting its movement toward the anode during electrophoresis. We focused the fluorimetric analysis on the baseline fluoresces to assess the hydrophobicity of MCAD variants, as thermal stability of these variants has been previously reported^25^. Additionally, from the MCAD WT crystallographic structure (PDB 1EGE) it seems likely that R206 forms a salt bridge with the aspartate at position 208, stabilizing a loop most probably required for proper folding of the beta-domain. Then, exchanging an arginine for a cysteine at position 208 would destabilize this loop. Moreover, the formation of a variant-specific disulfide bond is unlikely given that the closest cysteine (C159) resides ca. 15 Å away, at the beginning of the beta-fold.

We have further characterized MCAD variants using alternative methodologies to evaluate the molecular mass and stoichiometry. By native MS, a mixture of two protein populations was observed, a predominant tetramer and a minor octamer, with tetramers being more abundant. The observation of two MCAD populations by native MS was not entirely consistent with hrCN gel separations, where an apparent homogeneous sample yielding one main band was detected. Therefore, the low intensity of native-MS octamer peaks at relatively high sample concentration suggests that these could be unspecific. Moreover, the difference in apparent molecular mass and stoichiometry between MCAD WT and the R206C variant observed by hrCN-PAGE could not be corroborated by the native MS analysis, where the calculated molecular masses of both, WT and R206C for tetramers was equivalent. This indicated that the aberrant migration pattern of variant R206C in hrCN gels was not due to a change in the stoichiometry or molecular mass of the protein, but be rather caused by a structural rearrangement of the MCAD tetramer, altering its shape or hydrophobicity, as this variant is predicted to cause a large conformational change^31^. Furthermore, analysis of the FAD redox state in recombinant variants showed fully reduced FAD in both K329E and R206, indicating that FAD redox state does not influence the separation pattern, as only variant R206C displayed abnormal migration despite similar FAD reduction. Importantly, native MS analysis of purified MCAD revealed binding of a molecule of 920 Da. Decanoyl-CoA was the best match for the Δmass and hence the most likely ligand. However, some differences at the FWHM are in line with presence of additional species such as octanoyl-CoA (894 Da). Interestingly, MCAD was not expressed, nor purified in the presence of MCAD substrates, suggesting that endogenous species from *E. coli* were incorporated during overexpression*.* Binding of the acyl-CoA may also have an effect on the protein redox state as well as on the interaction with the ETF complex, the physiological electron acceptor of MCAD.

Altogether, these findings indicate that the enzymatic activity measured by our in-gel colorimetric assay predominantly corresponds to the MCAD homotetrameric species, whose migration behaviour in native gels diverges from the expected typical pattern due to structural or conformational influences.

In summary, the in-gel assay used for determining the enzymatic activity of MCAD is capable to distinguish between varying conformations that may result from pathogenic variants. The in-gel assay described here can be adapted to determine the activity of other acyl-CoA dehydrogenases by replacing the substrate to match their specificity to further investigate the effects of genetic variants in other members of the acyl-CoA dehydrogenase family.

## Experimental procedures

### Expression and purification of human recombinant MCAD variants and ACADs.

Expression plasmids encoding the wild type (WT) *ACADM* or the MCAD variants K329E, R206C and Y67H were used for eukaryotic and prokaryotic expression. The previously established expression plasmid pMALc2X^17^ was used to clone *ACADM* with an N-terminal Tobacco Etch Virus (TEV)-protease cutting sequence, where mutations were included by site-directed mutagenesis. Plasmids for eukaryotic expression were previously described^17^. All plasmid inserts were verified by sequencing. To purify wild type and variant MCAD proteins, expression was induced in *E. coli* BL21-CodonPlus as described earlier^17^ with slight modifications. Briefly, cells were induced with 0.5 mM ITPG at 28 °C for 20 h in the presence of 1 mg/l riboflavin. MCAD protein tagged with maltose binding protein (MBP) was first purified from the cell lysates using an MBPTrap affinity chromatography column (GE Healthcare). After digestion with TEV protease, the column eluent was separated by size exclusion chromatography using a Superdex 200 gel filtration column to isolate the tetrameric non-tagged protein fraction. All steps were carried out in an ÄKTApure purification system at 4 °C. Absorbance spectra of MCAD variants is provided in Supplementary Fig. S1, where the FAD redox state was assessed by the absorbance ratio at 278/450 nm. A similar procedure was applied to purify wild type GCDH, SCAD and IVD.

### Overexpression of MCAD variants in COS-7 cells

African green monkey kidney COS-7 cells (ATCC CRL-1651) were cultured in RPMI 1640 medium (Gibco) supplemented with 10% foetal bovine serum (Capricorn Scientific) and 1% antibiotic–antimycotic solution (Corning) in a humidified incubator at 37 °C and 5% CO_2_. The absence of mycoplasma contamination was tested routinely. Transient expression of wild type *ACADM* (NM_000016.6, NP_000007.1) and variants was performed as described before^25^. Briefly, 1.25 × 10^5^ cells/well were transferred to a 96-well plate and transfected with 0.62 µg of DNA/well using the Amaxa electroporation system (Lonza). After transfection, cells were cultured for 48 h in a 96-well plate and washed once with PBS and 40 µl of 200 mM NaCl, 20 mM HEPES, pH 7.4 were added. Cells were disrupted by three freezing/thawing cycles. In the last cycle, 0.1% n-dodecyl-b-D-maltoside was added and cell suspensions were mixed by pipetting up and down. Cells from 16 to 24 wells were collected and centrifuged at 4000 g for 10 min at 4 °C. The supernatants were concentrated ~ 10 times by filtration through Amicon 10 kDa filters (Merck) and protein concentration was determined by Lowry.

### Native gel electrophoresis

Protein solutions of human recombinant MCAD variants (ca. 3 mg protein/ml) were thawed on ice and diluted to 1 mg/ml with ice-cold 0.5 M 6-aminohexanoic acid, 1 mM EDTA, 50 mM imidazole/HCl, pH 7.0 and separated by blue-native (BN-) or high-resolution clear native (hrCN-) polyacrylamide gel electrophoresis (PAGE) as described before^32^. Gradient polyacrylamide gels (4–16% or 4–20%) were prepared with Rotiphorese Gel 40 (Carl Roth), 0.1 g glycerol/ml, 15 mM imidazole, 0.5 M 6-aminohexanoic acid, pH 7.0. The composition of the anode buffers was 25 mM imidazole/HCl pH 7.0. For the cathode buffer, we used and 50 mM tricine, 7.5 mM imidazole, pH ~ 7.0, supplemented with 0.02% Coomassie Brilliant Blue (G-250, Serva) for BN-PAGE or with 0.05% sodium deoxycholate and 0.01% n-dodecyl-β-D-maltoside for hrCN-PAGE, according to previous procedures^19^. Gels were run, first, at 100 V and after 15 min, the voltage was increased to 400 V and continued to run for 2–3 h. NativeMark unstained protein standards (Invitrogen) were used for molecular mass calibrations. For testing the effect of the ionic strength on protein migration, 6-aminohexanoic acid was omitted from the gel formulation.

### FAD content determination

FAD contains an isoalloxazine ring that, in the oxidized state, emits fluorescence at ~ 515 nm wavelength when excited with blue light^33^. For quantification of the FAD content in MCAD protein variants, after electrophoresis, only clear gels were documented directly in a ChemiDOC TC20 (Bio Rad Laboratories) by excitation with blue light using the standard settings for Alexa Fluor 488 dye to determine the intrinsic fluorescence of FAD. Due to interference with Coomassie blue, it was not possible to determine the FAD content from blue native gels.

### In-gel activity determination

After electrophoresis, blue and clear gels were immediately immersed in the reaction mixtures to perform the enzymatic colorimetric reactions. The reaction mixture contained 0.5 mg/ml nitroblue tetrazolium chloride, 0.2 mM phenazine methanosulfate and 5 mM Tris, pH 7.4, and 100 µM of the acyl-CoA substrate, either octanoyl-CoA (C8-CoA), or phenylpropionyl-CoA (PP-CoA) were added to start the reaction. The gels were incubated at room temperature with constant agitation and the reaction was stopped with 50% methanol, 10% acetic acid. The gels were washed with distilled water and documented on a ChemiDOC TC20 using the standard settings for Coomassie blue staining. In the case of human recombinant MCAD variants, purple bands corresponding to the activity of MCAD oligomers started to appear already after 10–15 min, but for reproducibility purposes, the reactions were stopped after 30 min. In the case of MCAD variants overexpressed in COS-7 cells, the gels were incubated in the reaction mixture for 1 h.

### Spectrophotometric activity determination

The activity of recombinant MCAD variants was determined spectrophotometrically as described before^14^ by following reduction of the artificial electron acceptor ferricenium hexafluorophosphate (Santa Cruz biotechnology) at 300 nm in a CLARIOstar microplate spectrophotometer (BMG Labtech). The reaction mixture contained 200 mM Tris, pH 8.0, 200 µM ferricenium hexafluorophosphate and 0.625, 1.25 or 2.5 µg protein/ml of the recombinant variants and was started with 100 µM octanoyl-CoA.

### Gel quantifications

After electrophoresis and the corresponding staining procedures, gels were documented in a ChemiDOC TC20. Images were cropped to highlight the bands corresponding to the proteins of interest. Uncropped images of all gels are shown in Supplementary Fig. S7. The protein bands were quantified using ImageJ v1.48 (imagej.org) and the integrals of the peaks were calculated in Origin 6.0 (OriginLab).

### Isoelectric focusing

To determine the isoelectric point of MCAD variants, 5 µg of protein were separated using Novex pH 3–10 IEF gels (Invitrogen, Thermo Fisher Scientific) and gels were stained with Coomassie blue. IEF-Marker 3–10 (Invitrogen) was used for calibration.

### Bottom-up proteomics analysis of hrCN-PAGE gel lanes

Fibroblasts were separated by hrCN-PAGE using 5–16% polyacrylamide gradient gels. After electrophoresis, gel was stained with Coomassie blue and entire gel lanes were cut into 60 even slices. Each gel slice was subjected to in-gel trypsin digestion and analysed by liquid chromatography tandem mass spectrometry as described before^34^. Mass spectrometry raw files were analysed using MaxQuant (v1.5.0.25) matching experimental spectra against the Uniprot human database. 24 MCAD unique peptides, corresponding to 52.5% sequence coverage, were used for reconstruction of the abundance-mass profiles of MCAD. Part of the results generated by this data set of protein groups has been published before^35^.

### Native mass spectrometry

Purified human recombinant MCAD at 4 mg/ml (91.66 µM of monomer) in 200 mM NaCl, 1 mM DTT, HEPES 20 mM, pH 7.4 was diluted to 0.8 mg/ml (18.33 µM) in ice-cold 200 mM ammonium acetate (Sigma-Aldrich, ≥ 98%), 1 mM DTT, pH 7.4 and re-concentrated using Microcon-10 kDa centrifugal filter units at 4 °C. This procedure was repeated six times until the NaCl concentration was negligible. MCAD solutions were analysed in triplicates in a Q Exactive UHMR (Thermo Fischer Scientific, Massachusetts, U.S.) or in a high-mass modified^36^ Q-TOF 2 time-of-flight mass spectrometer (Waters, Manchester, UK and MS Vision, Almere, the Netherlands) as described previously^37^. To spray the sample into the instrument, gold-coated borosilicate glass capillaries, were prepared. Borosilicate glass tubes with an inner diameter of 0.68 mm and an outer diameter of 1.2 mm with filament (World Precision Instruments, Novato, CA, USA) were pulled into fine capillaries using a two-loop program on a micropipette puller (Sutter Instruments, Novato, CA, USA) containing a squared box filament (2.5 × 2.5 mm). Afterwards the capillaries where gold-coated on a Safematic sputter coater (Zizers, Switzerland; process pressure 5 × 10^−2^ mbar, process current 30.0 mA, coating time 100 s, 3 runs to vacuum limit 3 × 10^−2^ mbar Argon). Proteins were then introduced into the UHMR spectrometer via a nanoESI source. The capillary voltage (1.45 kV) and cone voltage (150 V) were kept steady during the measurement. To avoid in-source collision induced dissociation (CID) acceleration voltage was kept at 10 eV. Acceleration voltage used in the high collision dissociation cell was altered during experiments to initiate dissociation of the proteins. As collision gas nitrogen (UHMR) or argon Q-TOF 2 were used at a pressure of 1.5 × 10^–2^ mbar. Prior to the experiments the instrument was calibrated with 25 mg/mL CsI in H_2_O. Spectra were later analysed via Xcalibur 4.2.47 (Thermo Fisher Scientific, MA, USA), mMass^38^ and Massign^39^.

### Size exclusion chromatography

Protein solutions were diluted in 200 mM NaCl, 1 mM DTT, HEPES 20 mM, pH 7.4 to 2 mg/ml and separated by size exclusion chromatography for native proteins in an Ultimate 3000 Dionex high-pressure liquid chromatography (HPLC) system (Thermo Fisher Scientific) with a Superdex 200 5/150 GL (Cytiva 28-9065-61) column operating at 4 bar with a flow of 0.1 ml PBS/min at 5 °C. Molecular mass standards (Gel Filtration HMW Calibration Kit, GE Healthcare) were diluted in PBS to reach a concentration of 2 mg/ml. Chromatograms were generated by monitoring absorbance at 280 nm in a Ultimate 3000 RS variable wavelength detector (Thermo). The molecular masses of MCAD protein variants were estimated based on a calibration curve using protein standards. To generate the calibration curve, the logarithms of the molecular masses of the standards were plotted as a function of the elution volumes where the elution peaks reached their maxima.

### Differential scanning fluorimetry

Differential scanning fluorimetry was performed following the fluorescence of Protein Thermal Shift Dye (446,114, Thermo Fisher Scientific) at 470 ± 15 nm and 623 nm ± 14 nm as excitation and emission wavelengths, respectively, in a Applied Biosystem QuantStudio 12 K Flex real-time PCR system. Fluorescence was determined from 20 to 85 °C increasing at a rate of 0.08 °C/s, measured in quadruplicates in a 384-well and a total volume of 20 µl. Reaction mixture contained 0.1 mg protein/ml, and 1:1000 dilution of the dye in 200 mM NaCl, 20 mM HEPES, pH 7.0. Fluorescence values from a control without protein were subtracted. Hydrophobicity of recombinant MCAD variants was assessed as the baseline fluorescence values in comparison to the WT.

## Supplementary Information

Supplementary Information.

## Data Availability

Protein groups data from the bottom-up proteomics analysis is available at the complexome profiling data resource CEDAR, (https://www3.cmbi.umcn.nl/cedar/browse/experiments), accession number: CRX46. Raw native MS data is available on request (Charlotte Uetrecht, charlotte.uetrecht@cssb-hamburg.de).
