# Supplementary material for: High-resolution native electrophoresis in-gel activity assay reveals biological insights of medium-chain fatty acyl-CoA dehydrogenase deficiency
Source: Sci Rep. 2025 Oct 23;15:37168. doi: 10.1038/s41598-025-24684-3 (PMC12550018; doi:10.1038/s41598-025-24684-3)
Supplement: Supplementary file 1 — Supplementary Information. [file 41598_2025_24684_MOESM1_ESM.pdf]

Supporting information

High-resolution native electrophoresis in-gel activity assay reveals biological insights of medium-chain fatty acyl-CoA dehydrogenase deficiency.

Sergio Guerrero-Castillo*, Alice Grün, Nicole Lewandowski, Polina Gundorova, Lisa Ela Blettenberger, Nora Constanze Laubach, Katrin Küchler, Madalena Barroso, Charlotte Uetrecht, Søren W. Gersting*.

* Correspondence:
Dr. Sergio Guerrero-Castillo
s.guerrerocastillo@uke.de
Prof. Dr. Søren W. Gersting
gersting@uke.de

Content:

Supporting Figures S1-S7
Supporting Tables S1 and S2

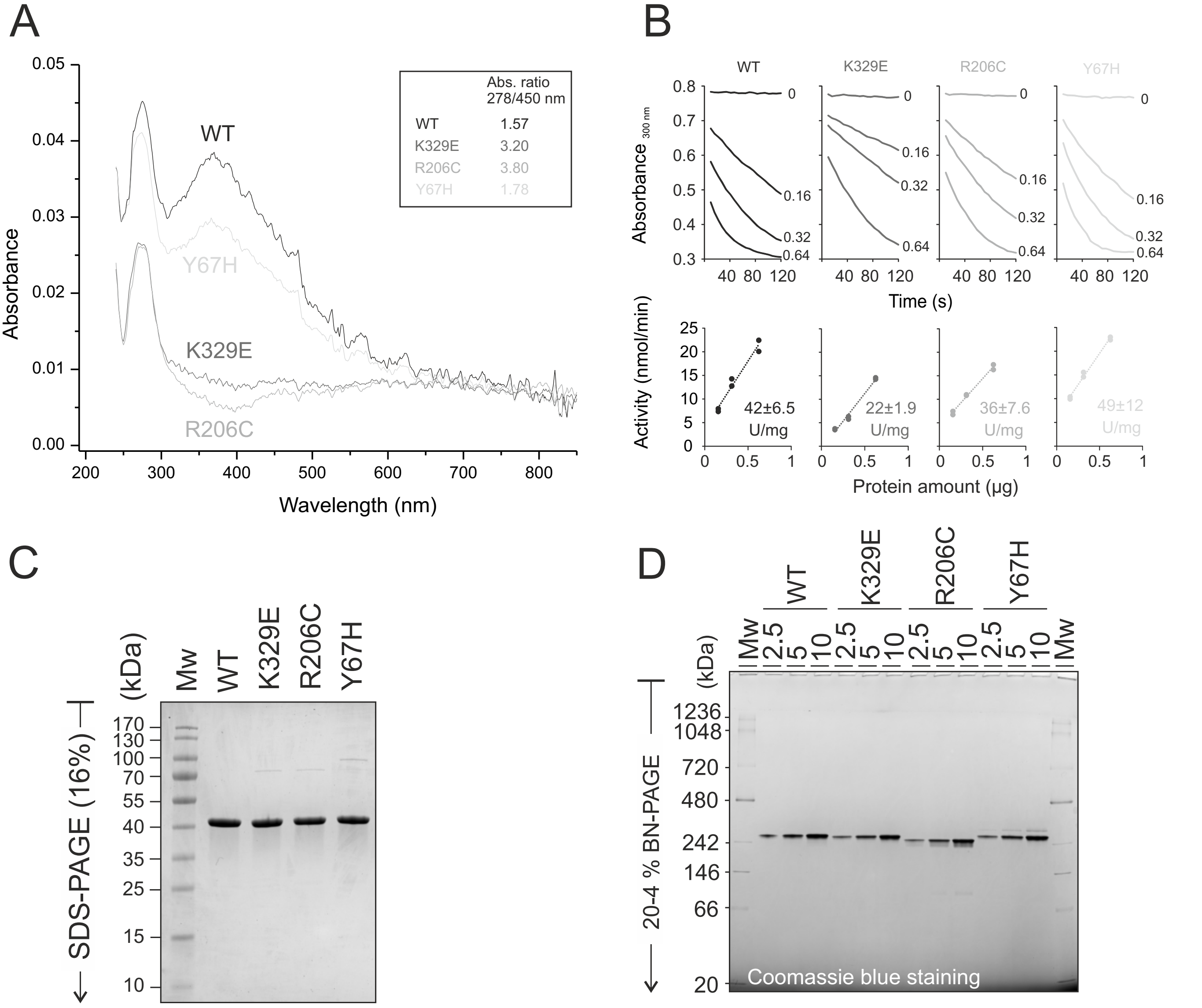

Supplementary Figure S1. Absorbance spectra, spectrophotometric activity and electrophoretic separation patterns of MCAD variants in denaturing and blue-native gels. A) Absorbance spectra of human recombinant MCAD variants. B) Spectrophotometric activity of recombinant MCAD variants following reduction of ferricenium hexafluorophosphate by octanoyl-CoA at 300 nm. Amount of protein (µg) is indicated next to each trace in the upper panel. Lower panel shows the activity for each MCAD variant determined by the initial slopes of each trace. Numbers indicate the means of the activity ± standard deviation in U/mg protein (n=4). C) purified human recombinant MCAD WT and variants separated by tricine-SDS-PAGE (3 µg protein/lane). D) human recombinant MCAD protein variants separated by BN-PAGE (Amount of protein in µg indicated above the gel lanes).

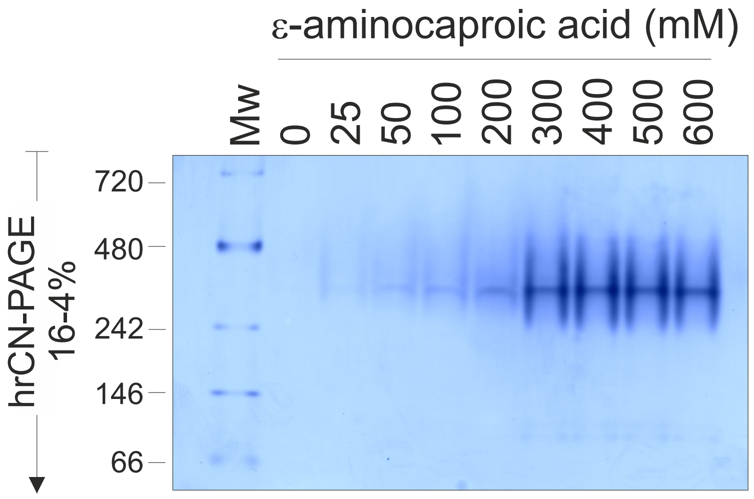

Supplementary Figure S2. Lack of surface charge effect on electrophoretic mobility of recombinant MCAD WT in hrCN gels. Purified human recombinant MCAD WT were diluted 1:3 in solubilization buffer with 1 mM EDTA, 50 mM imidazol, pH 7.0 and the indicated concentrations of aminocaproic acid. 6 µg protein/lane were loaded on a 4-16% clear native gel without aminocaproic acid in the gel formulation.

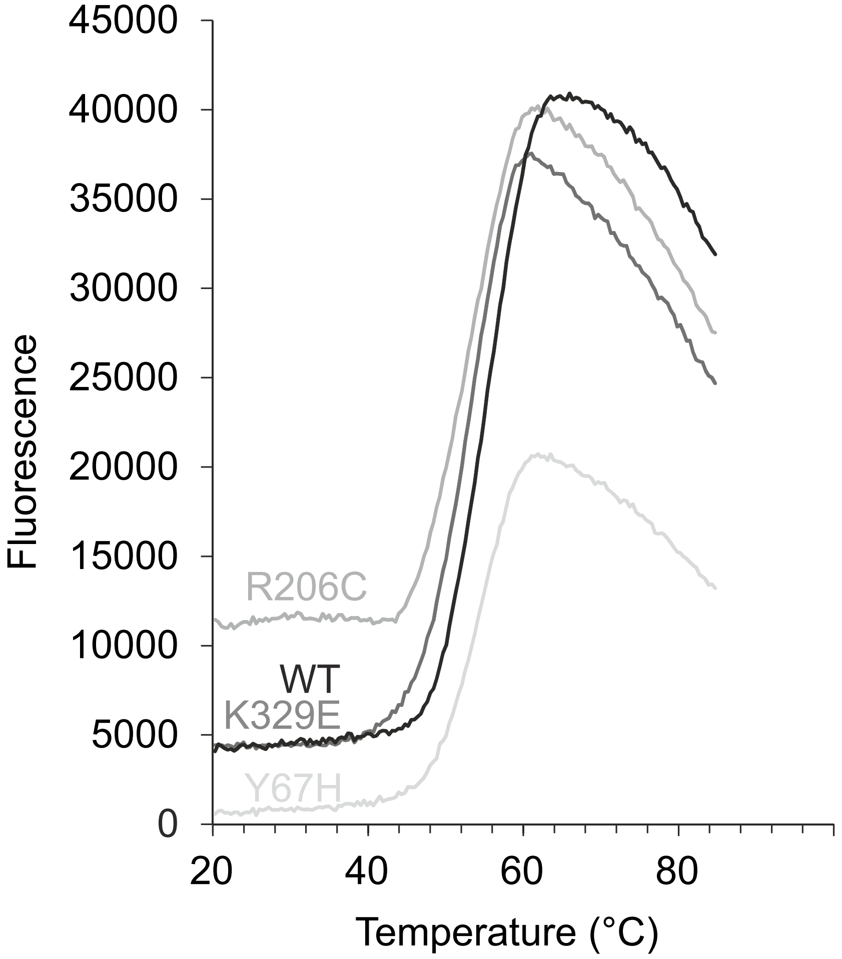

Supplementary Figure S3. Hydrophobiciy of MCAD variants assessed by differential scanning fluorimetry. Purified human recombinant MCAD variants were diluted to 0.1 mg/ml in 200 mM NaCl, 20 mM HEPES, pH 7.0 and mixed with a 1:1000 dilution of Protein Thermal Shift Dye. Baseline fluorescence (between 20 and 40 °C) was used to indirectly evaluate hydrophobicity of MCAD variants. Average traces of four replicates are shown.

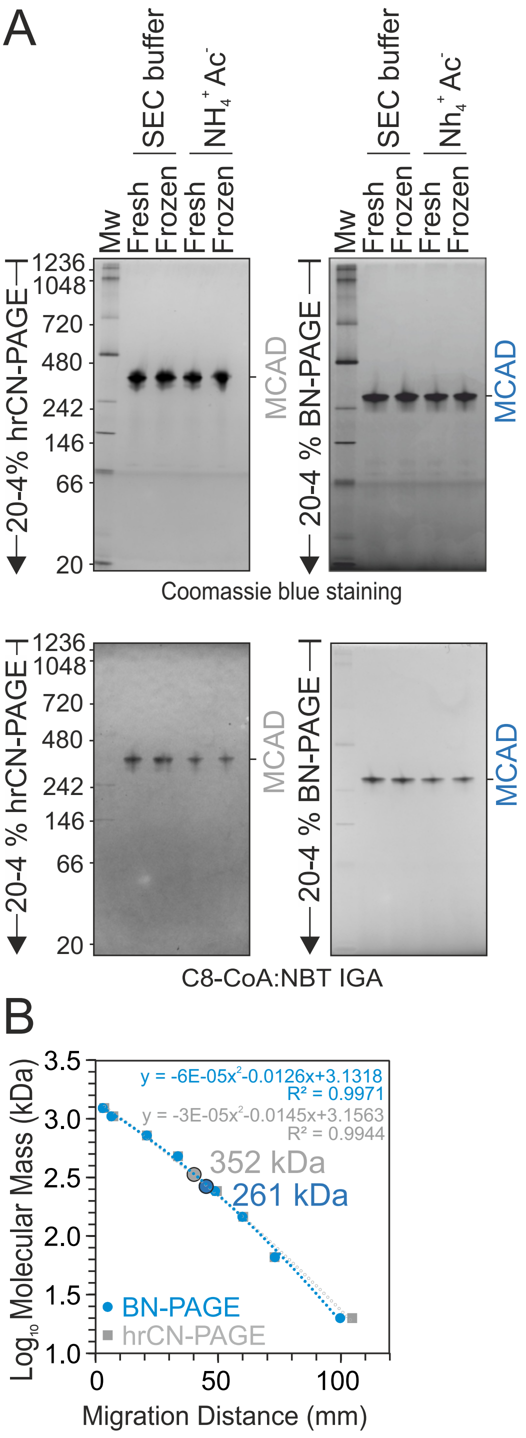

Supplementary Figure S4. Stability of MCAD oligomers in ammonium acetate buffer. A) Buffer replacement test. Separation of MCAD WT by BN-PAGE (left panels) or hrCN-PAGE (right panels) with or without replacing SEC buffer with ammonium acetate buffer, and with or without a freezing/thawing cycle. Coomassie blue staining (upper panels) and C8-CoA:NBT in-gel activity staining (lower panels) are shown. B) Molecular mass calibration and interpolation of MCAD predominant bands after separation by BN- and hrCN-PAGE.

Supplementary Table 1. Deconvoluted masses and peak resolution of MCAD tetramers in charge state +26.

	Deconvoluted Mass (Da)					FWHM				
**MCAD Variant**	**Peak 1**	**Peak 2**	**Peak 3**	**Peak 4**	**Peak 5**	**Peak 1**	**Peak 2**	**Peak 3**	**Peak 4**	**Peak 5**
WT	178010 ± 20	178930 ± 30	179850 ± 30	180770 ± 30	181690 ± 30	130 ± 50	130 ± 40	140 ± 30	150 ± 30	160 ± 60
K329E	177995 ± 3	178913 ± 5	179835 ± 8	180760 ± 20	181667 ± 8	100 ± 30	120 ± 30	150 ± 70	130 ± 20	200 ± 200
R206C	177000 ± 10	177790 ± 9	178710 ± 20	179630 ± 20	180570 ± 20	120 ± 20	130 ± 20	140 ± 30	160 ± 30	120 ± 90
Y67H	177890 ± 30	178810 ± 30	179720 ± 30	180640 ± 30	181550 ± 30	100 ± 30	120 ± 20	130 ± 20	140 ± 20	160 ± 60

Supplementary Table 1. Determined masses from native MS. Average masses in Da and associated error (standard deviation) from triplicate measurements are provided for unoccupied (0) and 1-4 bound decanoyl-CoA. FWHM (full width half maximum) in Da of the peaks is also given, which is clearly below the masses of the bound cofactors in line with high confidence assignments.

Supplementary Table 2. Analysis of molecular masses of MCAD tetramers.

**MCAD Variant**	**Theoretical mass (Da)**	**Mass difference from unoccupied to theor. mass**	**Mass difference between peaks in charge state +26**			
WT	174800	3210	920	920	920	920
K329E	174804	3191	918	922	925	907
R206C	174588	2412	790	920	920	940
Y67H	174696	3194	920	910	920	910

Supplementary Table 2. Examination of mass differences between experimental and theoretical masses. The difference between theoretical and unoccupied measured masses corresponds to four or three bound FAD cofactors. Most mass differences from unoccupied to 1 bound peak correspond to decanoyl-CoA, except for R206C, where this peaks marks the fourth FAD bound with lower occupancy followed by binding of up to 3 decanoyl-CoA.

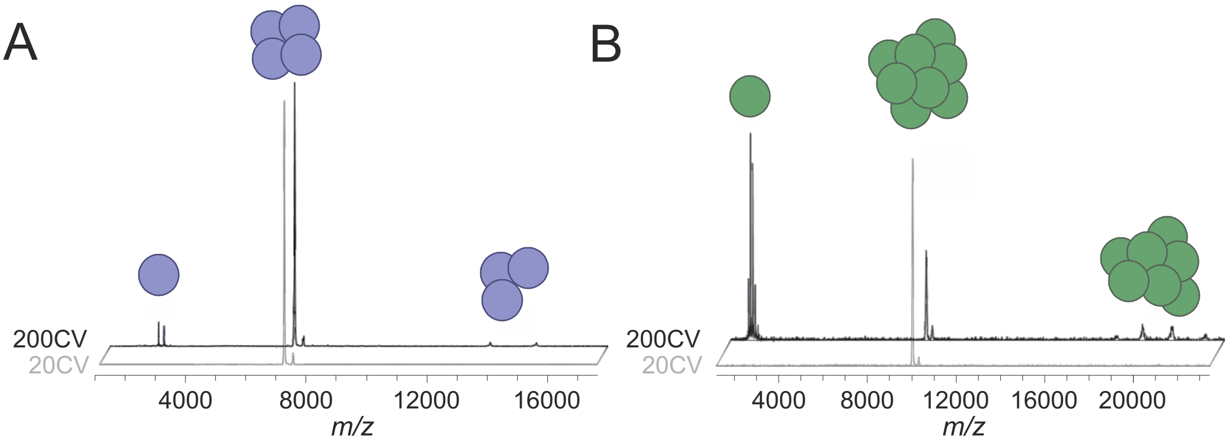

Supplementary Figure S5. MS/MS of human recombinant MCAD wild type. Tandem mass spectrometry analysis of the MCAD tetramer (A) and octamer (B). The tetrameric and octameric species at *m/z* ~7250 and ~10100 were isolated and dissociated using high collision energy. Ejection of monomer is observed. The resulting trimeric (A) and heptameric (B) high mass complexes confirm the assignment of the oligomeric state of the precursors.

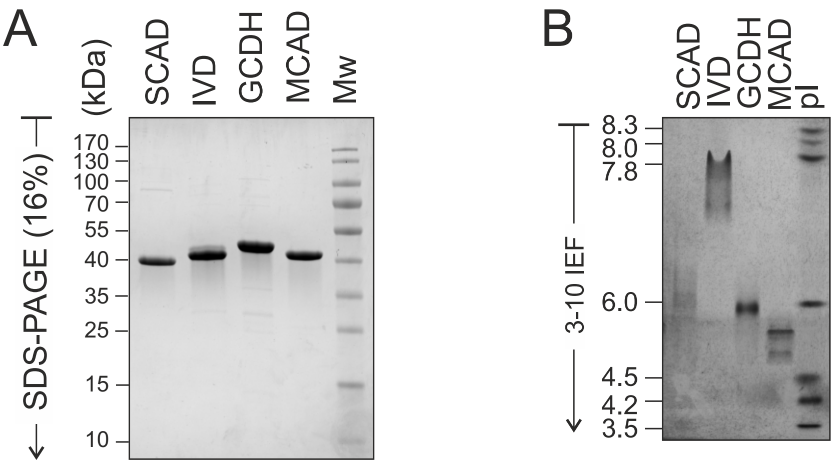

Supplementary Figure S6. Electrophoretic separation patterns of ACADs in denaturing gels and isoelectric focusing. A) purified human recombinant WT acyl-CoA dehydrogenases separated by tricine-SDS-PAGE (3 µg protein/lane). B) Isoelectric focusing of ACADs using 3-10 IEF gels followed by Coomassie blue staining.

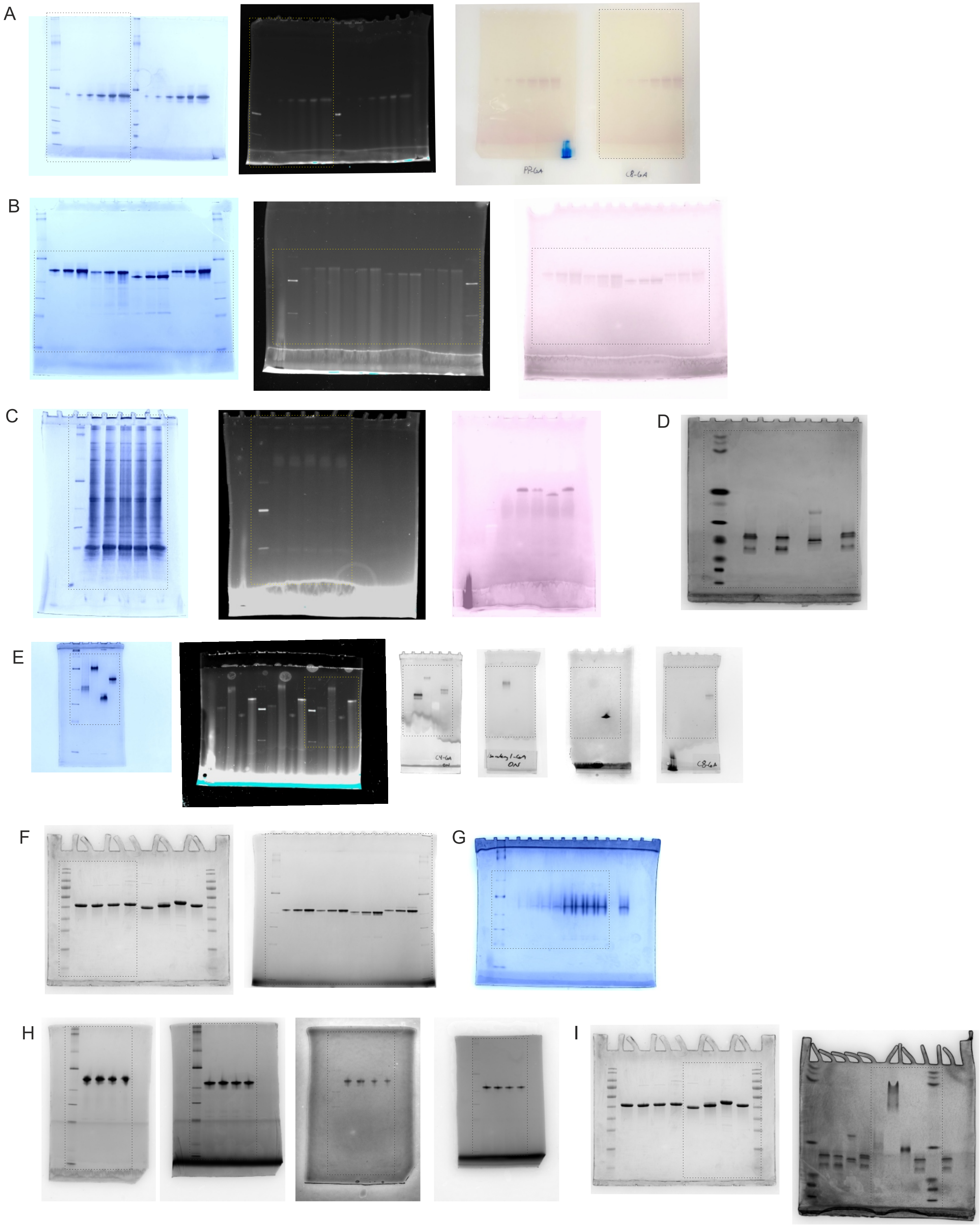

Supplementary Figure S7. Full-length images of gels shown in all figures. Uncropped images of gels shown in Figure 1 (A), Figure 2 (B), Figure 3 (C), Figure 4 (D), Figure 6 (E), Figure S1 (F), Figure S2 (G), Figure S4 (H) and Figure S6 (I). Cropped areas are indicated.
